# From Waste to Added-Value Product: Synthesis of Highly Crystalline LTA Zeolite from Ore Mining Tailings

**DOI:** 10.3390/nano13081295

**Published:** 2023-04-07

**Authors:** Jhuliana Campoverde, Diana Guaya

**Affiliations:** Department of Chemistry, Universidad Técnica Particular de Loja, Loja 110107, Ecuador

**Keywords:** mining tailing, hydrothermal synthesis, LTA, sodalite, methylene blue, lithium, nanoparticles

## Abstract

The use of wastes is necessary to contribute to environmental sustainability. In this study, ore mining tailings were used as the raw material and precursor for the synthesis of LTA zeolite, a value-added product. Pre-treated mining tailings were submitted to the synthesis stages under specific established operational conditions. The physicochemical characterization of the synthesized products was performed with XRF, XRD, FTIR and SEM, to identify the most cost-effective synthesis condition. The LTA zeolite quantification and its crystallinity were determined as effects of the SiO_2_/Al_2_O_3_, Na_2_O/SiO_2_ and H_2_O/Na_2_O molar ratios used, as well as the influence of the synthesis conditions: mining tailing calcination temperature, homogenization, aging and hydrothermal treatment times. The zeolites obtained from the mining tailings were characterized by the LTA zeolite phase accompanied by sodalite. The calcination of mining tailings favored the production of LTA zeolite, and the influence of the molar ratios, aging and hydrothermal treatment times were determined. Highly crystalline LTA zeolite was obtained in the synthesized product at optimized conditions. Higher methylene blue adsorption capacity was associated with the highest crystallinity of synthesized LTA zeolite. The synthesized products were characterized by a well-defined cubic morphology of LTA zeolite and lepispheres of sodalite. The incorporation of lithium hydroxide nanoparticles over LTA zeolite synthesized (ZA-Li^+^) from mining tailings yielded a material with improved features. The adsorption capacity towards cationic dye was higher than for anionic dye, especially for methylene blue. The potential of using ZA-Li^+^ in environmental applications related to methylene blue deserves detailed study.

## 1. Introduction

Mining is a key economic activity because it is the origin of many materials and energy sources used for the large-scale production of globally marketed products. Mining is based on the exploitation of minerals that produce wastes known as tailings [1]. Conventionally, mine tailings are stored without technical or environmental criteria. Currently, several mining waste hills are located near towns. The tailings are composed of fine-grained material and variable water content, which facilitates the dissolution of toxic substances. The increase in heavy metals and the generation of sulfuric acid cause risks of contamination for soil and groundwater. The existence of pollutants drastically affects the health of animals, plants and people in surrounding towns [2]. Tailings are toxic by-products of mining processes and mineral concentration. They are a mixture of soil, water, minerals and rocks, and contain high concentrations of chemicals and elements that pollute the environment. Thus, mining tailings require appropriate transport and storage for later treatment and reuse [3].

In recent decades, mining waste amounts have grown in large proportion due to the increase in annual extraction and processing of mineral resources [4]. According to reports, between 1 and 3% of the waste concentrated from the total ore processed from 97 to 99% of solid tailings. It is estimated that between 5 and 7 billion tons of mine tailings are generated annually worldwide [5]. In Latin America, particularly in Ecuador, about 167 mining environmental liabilities, including mining galleries, sanitary landfills, tailings deposits and mineral processing plants in mining areas require intervention. They were associated with potentially toxic elements that require environmental management and pollution mitigation [6]. Particularly, southern Ecuador is affected by serious technical problems regarding the construction of tailing deposits characterized by unstable platforms, leaching problems and damage of drainage systems, as well as their visual and landscape impacts [7].

The circular economy promotes the reuse and recycling of mine tailings as raw materials, contributing to environmental sustainability and the efficient use of natural resources. The reuse of wastes also provides cost-effective reagents for processes with environmental benefits, becoming value-added products [8]. In China, the reuse of bauxite tailings for the synthesis of zeolite type A was studied [9]. In Brazil, the use of mine tailings as raw material for the synthesis of zeolites was reported for wastewater treatment [10]. Mining tailings are used as a precursor for zeolite synthesis as a value-added product, and they can be used in the environmental remediation field as a material for efficient water pollution control. Zeolites have good physicochemical properties such as water stability, high specific surface area and excellent adsorption properties. Zeolites are porous materials conventionally used in wastewater treatment applications as an adsorbent or catalyst for the removal of pollutants, nutrients, organic dyes and heavy metal ions [11].

Zeolites are crystalline aluminosilicate materials characterized by a framework containing water and alkaline cations [12]. Si or Al atoms are placed in the center and O_2_ atoms in the corners, shared between S_i_O_4_ and AlO_4_ [13]. Zeolites develop good and unique properties; they have a low density and large volume of free space due to their specific structure. Their selectivity and high degree of hydration and crystallinity are also properties that make zeolites an optimal adsorbent, ion exchanger and catalyst [14]. Zeolites are synthesized using diverse methods and raw materials. Slight changes in the synthesis conditions such as the chemical composition of the reaction, synthesis time, reaction temperature and the types of directing agents, can produce different crystalline phases [15]. Zeolite Lynde type A (LTA) is produced commercially using the aluminosilicate hydrogel route from pure chemical precursors: silica and aluminum-based materials. Synthetic zeolite LTA is preferred due to its particle size homogeneity and high crystallinity, in comparison with natural zeolite [16].

Previous reports established the synthesis of LTA zeolite using a hydrothermal method from mining tailings [10,17]. The synthesis of LTA zeolites consists of four stages: alkali fusion, hydrothermal treatment, washing and drying [18]. The hydrothermal method with alkaline fusion uses a mixture of Si and Al species, metal cations, organic molecules and water, which are converted through a supersaturated alkaline solution and influenced by high temperatures into a microporous crystalline aluminosilicate [19]. Hydrothermal synthesis has many advantages in comparison with other methods; it has low energy requirements and low pollution, where the formation of a unique metastable and condensed phase is favored due to the increase in reactivity from reagents, and the solution can be easily controlled [20]. Previous reports demonstrated that the experimental conditions and molar ratios (SiO_2_/Al_2_O_3_, Na_2_O/SiO_2_ and H_2_O/Na_2_O) determine the type of zeolite synthesized and the quantification of the synthetic products [17].

LTA zeolite is a common adsorbent of pollutants, and it is well known that the support of metal nanoparticles improves its adsorption capacity [21]. The activation of LTA zeolite substituting sodium ions by others increases the affinity between adsorbates and zeolites, such as it has been used with lithium [22]. Lithium-aluminum-layered double hydroxides have been widely reported for adsorption applications [23,24,25]. Although lithium has been used for the preparation of some materials with abilities to remove pollutants, e.g., adsorption of heavy metals [26], there is little information about lithium nanostructures being developed over LTA zeolite for adsorption applications. Most of lithium materials have been obtained from lithium-ion battery wastes [27], but the development of lithium nanostructures demonstrated better performance for adsorption applications [28]. In this study, dyes were selected as the study case, due to their widespread use in industry for coloring products, and their further presence in wastewater discharges, with adsorption being the most common technology used for dyes removal [29]. 

To the best of our knowledge, reports about the synthesis of LTA zeolite from low-cost silica–alumina sources lack information about the content and crystallinity of the obtained zeolite phases, and their implications for adsorption applications. Therefore, the novelty of this study is the production of highly crystalline LTA zeolite, which was synthesized from mining tailings. The crystallinity and quantification of the LTA zeolite phase were evaluated based on the operating conditions of the synthesis process. The relationship between the crystallinity of the synthesized zeolites and the removal of pollutants was also studied, evaluating dye removal as an environmental application. Finally, the support of lithium hydroxide nanoparticles over LTA zeolite is reported for the first time, as a promissory improvement in the adsorption properties of LTA zeolite. 

The objectives of this study were as follows: (i) to evaluate the influence of operation variables, such as calcination of raw mining tailings, molar ratios, aging and hydrothermal treatment times, on the synthesis of highly crystalline LTA zeolite; (ii) to evaluate the adsorption performance of synthesized zeolites as a function of the crystallinity and content of LTA; (iii) to support lithium hydroxide nanoparticles over the synthesized LTA zeolite; and (iv) to examine the adsorption viability of lithium hydroxide LTA zeolite towards dyes methylene blue, rhodamine B (cationic) methyl orange and tartrazine (anionic).

## 2. Materials and Method

### 2.1. Materials

Samples were obtained from a mining tailing dam at Relavera El Tablón (−3°43′47.87″ S, −79°37′15.44″ W), located in Portovelo (El Oro, Ecuador). The reagents used in this study included NaOH (EMSURE^®^ brand, Merck KGaA, Darmstadt, Germany) and NaAlO_2_ (Sigma-Aldrich, St. Louis, MO, USA), methylene blue hydrate, and tartrazine (Sigma-Aldrich. USA), And methyl orange and rhodamine B (LOBA Chemie PVT. LTD. Mumbai, India). All of the chemicals used in the test were analytically pure. 

### 2.2. Pre-Treatment of Mining Tailings

The mining tailings were pre-treated with a reduction in particle size. The sample was crushed in a Retsch RS 200 model (Haan, Germany) at 700 rpm/5 min. The sample was passed through a 200 mesh (75 μm) sieve to obtain homogeneously sized particle material. 

### 2.3. LTA Zeolite Synthesis

LTA zeolites were synthesized by an adaptation of the method reported by Zhang [17]. Specific values of SiO_2_/Al_2_O_3_, Na_2_O/SiO_2_ and H_2_O/Na_2_O molar ratios were established for the synthesis assays. The calcination of mining tailing, aging and hydrothermal treatment times were also considered as critical variables for the LTA synthesis. Two parts of deionized water were used to prepare a solution from NaOH + NaAlO_2_. A total of 5 g of mining tailings was mixed with the solution of NaOH + NaAlO_2_ and stirred for 30 min. The mixture was treated with alkali fusion for 4 h at 800 °C. The solid obtained was grounded and mixed with the remaining third of deionized water. The mixture was submitted to aging at room temperature, and was furthermore hydrothermally treated at 90 °C for the crystallization of LTA zeolite. The obtained LTA zeolite was washed until the pH decreased to between 7 and 8. The sample of synthesized LTA zeolite was dried at 90 °C and stored for further assays.

### 2.4. Incorporation of Lithium Hydroxide Nanoparticles

The incorporation of lithium hydroxide was performed using an adaptation of the method described in the literature [12]. Then, 30 g of the LTA zeolite sample was treated in 250 mL of LiCl (0.1 M) at pH 10 (by continuous addition of NaOH) for 6 h by reflux and constant stirring. The modification process was performed two times. The sample was washed until no chlorides were determined in the exhausted solution. The obtained ZA-Li^+^ was dried and calcined at 200 °C for 4 h.

### 2.5. Physicochemical Characterization of Materials

The chemical composition of the synthesized zeolite LTA samples were analyzed using X-ray fluorescence (XRF) with a Bruker model S1 Titan 800 handheld XRF analyser (Bruker, Billerica, MA, USA). Samples of synthesized zeolite LTA were analyzed using a X-ray diffractometer (XRD) (D8 Advance A25, Bruker, Karlsruhe, Germany). The diffraction patterns were acquired with a 2θ range between 4° and 50° using a Cu Kα anode radiation source (λ = 0.1542 nm) operating at 40 kV and 40 mA. The morphology of the samples was observed by scanning electron microscopy (SEM) using a field emission Tescan Mira 3 scanning electron microscope (Brno, Czech Republic). The nitrogen adsorption method was used for specific surface area determination in an automatic adsorption analyzer. Gas flow was established at 35:65% Ne:He, and it used the single-point method in a Micrometrics Chemisorb 2720 (Micrometrics Chemisorb, Norcross, GA, USA). The infrared absorption spectra were recorded between 4000 and 500 cm^−1^ using a Fourier Transform FTIR spectrometer at a frequency line of 50–60 Hz (Nicolet iS10, 4100 Jasco, Easton, MD, USA). To determine the mechanisms involved in lithium incorporation over the synthesized LTA zeolite, the lithium solution was analyzed in its initial and exhausted state. An Atomic Absorption Spectrometer AAA (Perkin Elmer, Waltham, MA, USA) and inductively coupled plasma optical emission spectroscopy (ICP-OES, Optima 8000 Perkin Elmer, Waltham, MA, USA) were used. The contents of Na, Fe, Ca, K and Zn were determined with the AAA; Li, Si and Al were analyzed with ICP-OES.

### 2.6. Study of the Adsorption Behaviour of the Synthesized Zeolites 

The adsorption performance of the synthesized zeolites was evaluated towards organic dyes methylene blue and rhodamine B (cationic), and methyl orange and tartrazine (anionic). Scanning in the broad wavelength regions was performed to determine the conditions at which each dye solution could be measured. The absorbances were measured at given wavelengths (λ) for methylene blue (664 nm), methyl orange (446 nm), rhodamine B (554 nm) and tartrazine (427 nm). Synthesized samples ZA1 to ZA8 (0.1 g) were equilibrated in an aqueous suspension of methylene blue (5 mg·g^−1^) for 24 h. A comparison of adsorption capacities was performed between synthesized parent sample ZA5 and ZA-Li^+^. Then, 0.1 g of ZA5 and ZA-Li^+^ were equilibrated for 24 h in an aqueous suspension of methylene blue (25 mg·g^−1^). Finally, ZA-Li^+^ (0.1 g) was equilibrated in aqueous solutions of each anionic and cationic dye (25 mg·g^−1^). The assays were performed by triplicate, and the average values were reported. The initial and equilibrium concentrations of each dye were determined, and their adsorption capacities were calculated according to Equation (1):

(1) $$Q_{e}=\frac{v\times (c_{o}-c_{e})}{w}$$

where Q_e_ is the equilibrium adsorption capacity (mg·g^−1^), v is the volume of dye solution (L), c_0_ and c_e_ are the initial and equilibrium dye concentrations (mg·L^−1^ PO_4_^3−^), and w is the mass of adsorbent used (g). 

## 3. Results

### 3.1. Characterization of Mining Tailings

The chemical composition of the mining tailings used in this study was determined by X-ray fluorescence (XRF) analysis (Table 1). SiO_2_ and Al_2_O_3_ were the major elements of the mining tailings, validating their use as a precursor for the synthesis of zeolite, which contains Si, Al and O_2_ in its structure [13].

The chemical composition of mining tailings before calcination at 800 °C revealed they were a dangerous raw material, limiting their application as a raw material for further processes. Their major components (e.g., SiO_2_ and Al_2_O_3_) were invariable; however, an important reduction in minority components (e.g., Cu, Zn, As and Pb) occurred after calcination. This can be explained in terms of heavy metals chelation to organic compounds; thus, they could be easily removed with organic matter by the effect of calcination [30]. In this study, the calcined mining tailings were below the allowable limit contents of heavy metals, according to the Canadian standard guidelines for sediment quality [Cu (18.7), Pb (35), Zn (123), As (5.9) and Hg (0.17)] [31]. 

**Figure 1:**
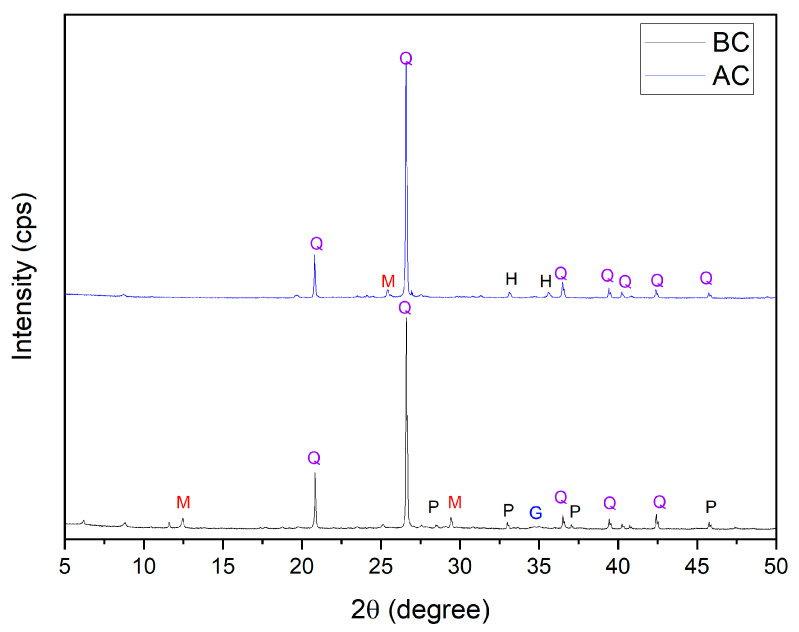
XRD patterns of mining tailings. BC: before calcination and AC: after calcination. (Q: quartz, M: muscovite, H: hematite, P: pyrite, G: geocronite).

The XRD patterns of the mining tailings, before and after being calcined, is depicted in Figure 1. The existence of characteristic reflections of quartz (Q), pyrite (P), geocronite (G) and muscovite (M) were determined according to standards reference codes No.1538064, No. 1534893, No. 9000139 and No. 1530033, respectively. Reflections of mining tailings before calcination indicated quartz (Q), pyrite (P), geocronite (G) and muscovite (M) as the main mineralogical phases. Quartz reflections were found at 2θ: 20.83° (100), 26.61° (101), 36.51° (110), 39.44° (102) and 42.42° (200). Reflections at 2θ: 28.50° (111), 33.01° (002), 40.26°(111) and 47.40° (202) revealed the presence of pyrite. Reflections of geocronite were observed at 2θ: 34.87° (110). Muscovite was observed at 2θ: 12.45° (002) and 29.43° (025). After the calcination of mining tailings, there were characteristic reflections found of one new crystalline phase: hematite (H) in agreement with the standard reference code No. 1526260. Reflections of hematite were detected at 2θ: 33.13° (104) and 35.60° (110). Reflections of quartz were found at 2θ: 40.24° (111), while for muscovite a new reflection was detected at 2θ: 25.58° (114). The crystallographic phases of mining tailings used in this study were comparable with those from other mining wastes previous reported [32]. The mining tailings after being calcined remained with characteristic reflections of quartz, muscovite and hematite. However, calcination promoted the release of heavy metals; thus, geocronite reflections were not detected, or were below the limit of quantification. Heavy metals chelated to organic compounds were easily removed by organic matter calcination [30].

### 3.2. Characterization of Synthesized Materials

Eight synthesized samples (SS1–SS8) allowed for optimal synthesis conditions for further samples (ZA1–ZA8). The operational conditions used in this study for the synthesis of LTA zeolite are summarized in Table 2. Mining tailing calcination, homogenization, aging and hydrothermal treatment times, and SiO_2_/Al_2_O_3_, Na_2_O/SiO_2_ and H_2_O/Na_2_O composition ratios were critical parameters used for the synthesis. The calcination of mining tailings allowed for the transformation of mineralogical phases that favored the LTA zeolitic phase. The variations in SiO_2_/Al_2_O_3_, Na_2_O/SiO_2_ and H_2_O/Na_2_O ratios had a significant effect for the production of amorphous products or other zeolite phases. The aging and hydrothermal treatment times also were responsible for the quantity and crystallinity of the synthesized LTA zeolites. The LTA crystallinity was determined according to Equation (2):

(2) $$\%\;\mathrm{Crystallinity}=\frac{\sum \mathrm{peak}\;\mathrm{areas}\;\mathrm{of}\;the\;\mathrm{synt}h\mathrm{esized}\;\mathrm{sample}}{\sum \mathrm{standard}\;\mathrm{sample}\;\mathrm{peak}\;\mathrm{areas}}\;\times \;100$$

#### 3.2.1. Chemical Composition of Synthesized Materials

The chemical composition of the synthesized zeolites determined by X-ray fluorescence (XRF) analysis are summarized in Table 3. SiO_2_ and Al_2_O_3_ were determined to be the main components of the zeolites.

The Si/Al ratio values of the eight synthesized samples were in the range between 1.08 and 1.24, which is in accordance with previous reports about LTA zeolite with a high cation exchange capacity and an Si/Al ratio ≅ 1 [33].

#### 3.2.2. Effect of SiO_2_/Al_2_O_3_, Na_2_O/SiO_2_ and H_2_O/Na_2_O Ratios on the Synthesis of LTA Zeolite

Conventionally, the synthesis of LTA zeolite has been established by the composition ratios from raw materials SiO_2_/Al_2_O_3_: 0.5–3.2, Na_2_O/SiO_2_: 1–2.5 and H_2_O/Na_2_O: 100–380 [17]. According to our previous research in synthesizing LTA zeolite from pure reagents, the following conditions were used as a starting point: SiO_2_/Al_2_O_3_: 3, Na_2_O/SiO_2_: 2 and H_2_O/Na_2_O: 40 [12]. Successively, many combinations of Al_2_O_3_, Na_2_O/SiO_2_ and H_2_O/Na_2_O ratios were used in this study. However, amorphous phases were obtained when the Na_2_O/SiO_2_ ratio was 1, while other unwanted phases of zeolite were obtained at an Na_2_O/SiO_2_ ratio of 3. For a low SiO_2_/Al_2_O ratio ≤ 3 and H_2_O/Na_2_O ≥ 170, amorphous phases were obtained. Other unwanted zeolitic phases were obtained when an SiO_2_/Al_2_O ratio above 3 and an H_2_O/Na_2_O ratio of 40 were used. The optimal ratios were found to be Na_2_O/SiO_2_: 2 and H_2_O/Na_2_O: 100, while SiO_2_/Al_2_O_3_: 8–13. A combination of LTA and sodalite zeolitic phases characterized samples ZA1 and ZA2, which was in accordance with previous reports about the coexistence of sodalite and LTA phases [34]. The content of sodalite for both ZA1 and ZA2 increased in comparison to the previous assay (SS8) when a reduction in the H_2_O/Na_2_O ratio occurred [33]; the aging time and the composition ratios were also critical.

**Figure 2:**
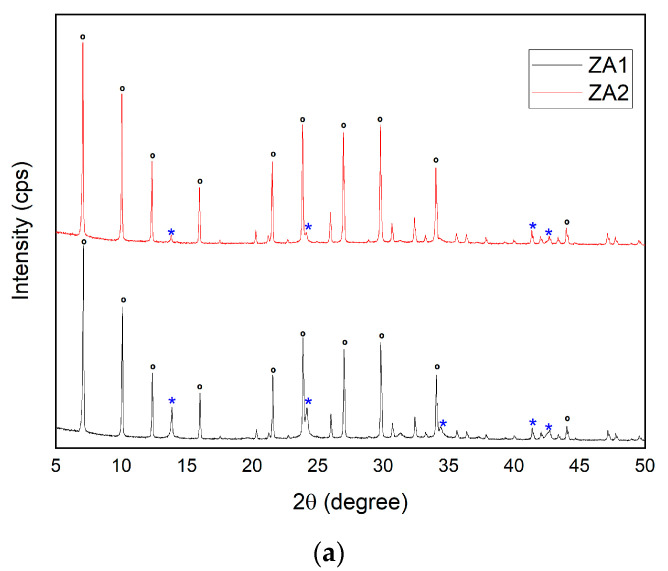

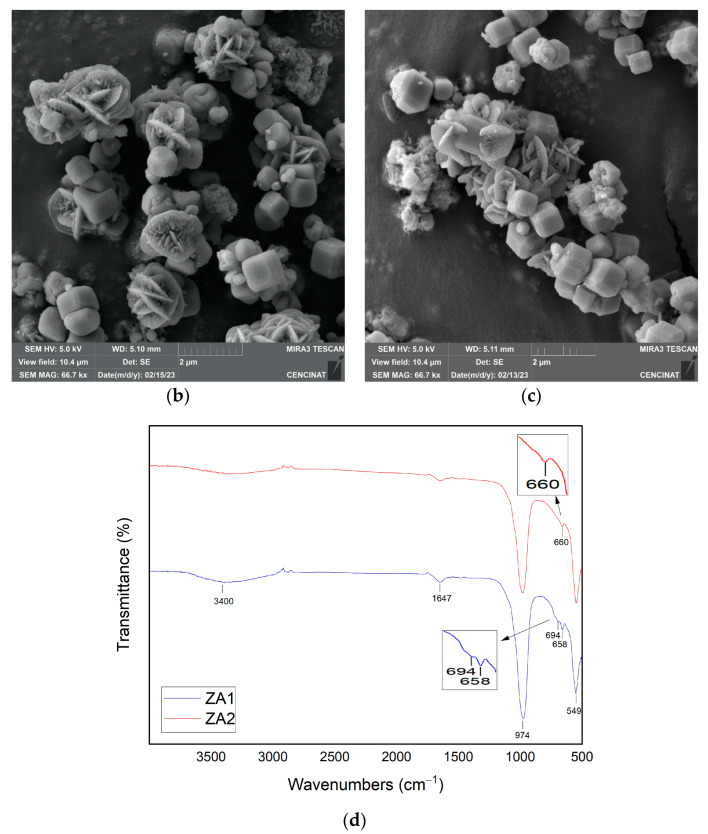
XRD patterns of synthesized zeolites ZA1 and ZA2 (**a**), * sodalite and **°** LTA. SEM micrographs of (**b**) ZA1 and (**c**) ZA2. FTIR spectra for ZA1 and ZA2 (**d**).

The XRD patterns of ZA1 and ZA2 are shown in Figure 2a. Sodalite and LTA zeolites were identified as the main mineralogical phases of samples ZA1 and ZA2. The existence of characteristic reflections of sodalite and LTA belong to the standards reference of the International Zeolite Association [35]. The main reflections of sodalite were detected at 2θ: 13.84° (011), 24.15° (112), 34.45° (222), 41.38° (088) and 42.55 (114) for ZA1; however, in ZA2, the reflection at 34.45° (222) was not found. Reflections at 2θ: 7.10° (002), 10.08° (022), 12.37° (222), 16.01° (024), 22.75° (026), 23.88° (226), 27° (246), 29.82° (028), 34.06° (466) and 44.02° (0012) revealed the presence of LTA zeolite for both the ZA1 and ZA2 samples. The peaks of LTA were indexed to the cubic crystal system and space group Fm-3c, with the refined unit cell parameters a[Å] = b[Å] = c[Å] = 24.55. The peaks of sodalite were indexed to the cubic crystal system and space group Im-3m, with the refined unit cell parameters a[Å] = b[Å] = c[Å] = 8.99. Higher intensity peaks of sodalite appeared when a low SiO_2_/Al_2_O_3_ ratio was used; ZA1 revealed higher intensity peaks than ZA2.

The SEM micrographs of samples ZA1 and ZA2 revealed a cubic morphology for LTA zeolite (Figure 2b,c). Heterogeneous particle sizes characterized the synthetized LTA zeolites, with ranges around 0.5–1.1 µm for ZA1 and 0.5–0.8 µm for ZA2. LTA crystals with well-defined edges were found in ZA2 in comparison to ZA1; this was in concordance with the higher crystallinity determined for ZA2. The formation of sodalite also occurred over the surface of LTA zeolite cubes, as was reported in previous studies [36]. The lepisphere morphology characteristic of sodalites [37] was determined, and heterogeneous particle sizes of sodalite ranged between 1.3 and 2.1 µm for ZA1, and between 1.0 and 1.7 µm for ZA2. 

The FTIR spectra for the ZA1 and ZA2 samples are represented in Figure 2d. The main absorption bands for both samples ZA1 and ZA2 were located at 549, 658, 694, 974, 1647 and 3400 cm^−1^. Bands at 549 and 974 cm^−1^ are typical features of zeolite structure. The absorption band at 974 cm^−1^ was associated with the vibration of the double ring in the TO4 tetrahedra [38]. The absorption band at 549 cm^−1^ was associated to the vibrations of sodalite cages, which are linked by double four-rings (D4Rs) [12]. The absorption bands at 3400 and 3600 cm^−1^ and 1647 cm^−1^ were due to intramolecular and intermolecular hydrogen bonds and intertwined interstitial water [39]. Finally, the absorption bands at 658 and 694 cm^−1^ corresponded to pure sodalite units, as reported in previous studies [40].

#### 3.2.3. Effect of the Reduction in Homogenization Time on the Synthesis of Zeolite LTA

The alkaline fusion treatment started once the water was added; the agitation at constant stirring speed was used for homogenization. Initially, 5 h of homogenization (ZA1 and ZA2) was used; however, a reduction in the homogenization time was performed to optimize the efficiency of synthesis process in terms of energy and time. Both samples ZA3 and ZA4 were obtained after 1 h of homogenization, but the content of LTA zeolite and its crystallinity were not strongly affected. Instead, the composition ratios were decisive for the synthesis of LTA zeolite with high content and crystallinity at specific aging and hydrothermal treatment times. ZA4 had higher LTA content and crystallinity than ZA3. 

**Figure 3:**
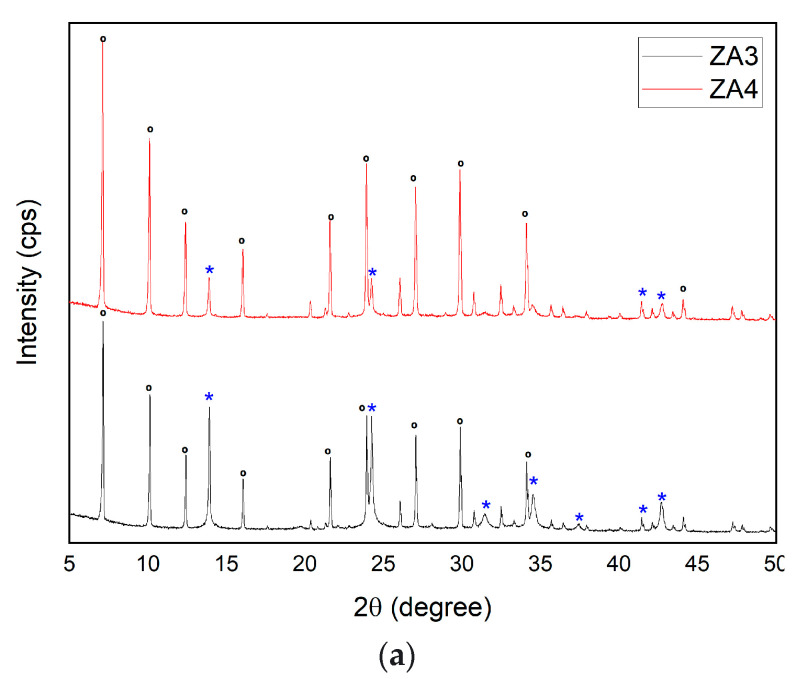

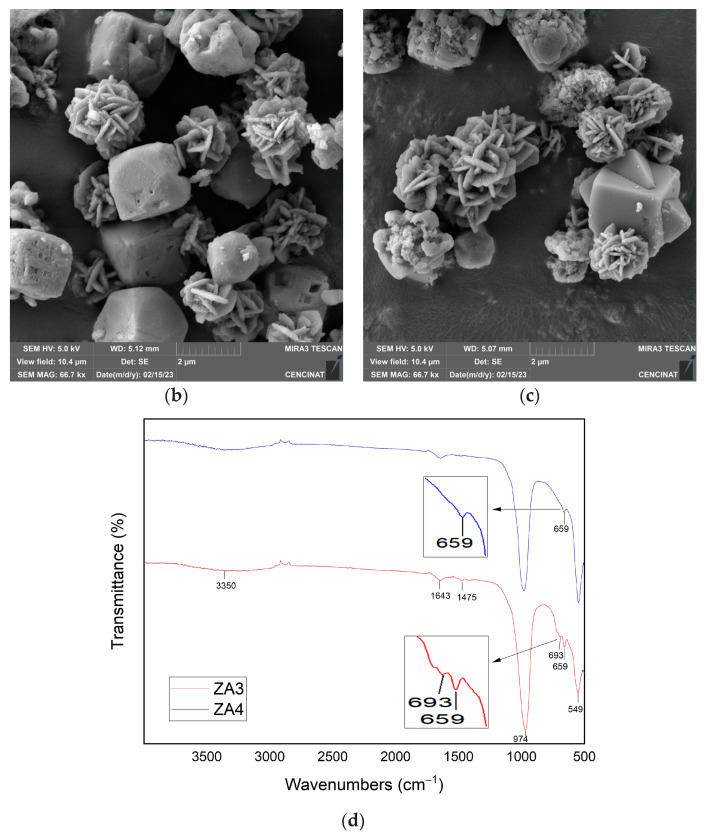
XRD patterns of synthesized zeolites ZA3 and ZA4 (**a**), * sodalite and **°** LTA. SEM micrographs of (**b**) ZA3 and (**c**) ZA4. FTIR spectra for ZA3 and ZA4 (**d**).

The XRD patterns of ZA3 and ZA4 (Figure 3a) demonstrated the existence of LTA and sodalite reflections in accordance with the standard reference of IZA. The LTA reflections were indexed to cubic crystal system and space group Fm-3c; however, a slight change occurred in the unit cell parameters a[Å] = b[Å] = c[Å] = 24.62. The peaks of sodalite were indexed to cubic crystal system and space group Im-3m, with a slight change in the unit cell parameters a[Å] = b[Å] = c[Å] = 8.97. Sodalite reflections were detected at the same 2q positions of ZA1, except that peaks at 2q: 31.46° (013) and 37.43° were observed in ZA3 but not in ZA4. In ZA3, the intensity of sodalite peaks was higher than that in ZA4, which is in accordance with the quantification of sodalite. The SiO_2_/Al_2_O_3_ ratio increased for the synthesized sample ZA4, and a decrease in the intensity of the sodalite peaks was observed; thus, a higher content of LTA and crystallinity were associated with sample ZA4. The negligible influence of agitation time for homogenization of the suspension during LTA synthesis was in accordance with the findings of a previous study, in which a gel remained without agitation between 5 and 24 h, which did not have a positive effect on zeolite crystallization [36].

Figure 3 reveals the characteristic morphology of the sodalite structures of both samples ZA3 (Figure 3b) and ZA4 (Figure 3c). Lepispheres were observed in ZA3 and ZA4 whose size oscillated around 1.3–2.1 µm. Sample ZA3 exhibited a poor crystalline cubic form of LTA zeolite with an average size of 0.8–1.8 µm for ZA3 and 1.1–2.1 µm for ZA4. However, the higher crystallinity of ZA4 was corroborated by the well-defined cubic morphology of LTA zeolite obtained [41], which was in accordance with the results of the XRD analysis.

The FTIR spectra for both samples ZA3 and ZA4 are represented in Figure 3d. The absorption bands at 659 and 693 cm^−1^ were associated with the sodalite content. ZA3 with its 64% sodalite content exhibited both bands at 659 and 693 cm^−1^; however, in ZA4 with 46% sodalite content, a unique band at 659 cm^−1^ was evidenced. ZA3 revealed a new band at 1475 cm^−1^ that has been associated with the hydroxy sodalite zeolite phase [42]; the low content of hydroxy sodalite is presumed since its typical petal-like aggregates morphology could not be easily distinguished and quantified by means of XRD analysis. During the initial reaction periods, LTA was obtained as a metastable zeolitic phase, but according to the Ostwald rule, the conversion of one zeolitic phase with low framework density into another with a higher framework density was possible. Precursor concentrations and reaction conditions promoted the successive transformation into a more thermodynamically stable zeolitic phase; thus, LTA transformed into sodalite and hydroxy sodalite [43,44].

#### 3.2.4. Effect of the Aging Time on the Synthesis of Zeolite LTA

The aging time at room temperature influenced the formation of the zeolite crystal nuclei and the crystallization rate [45]. In this study, the effect of varying the aging time was evaluated, which ranged between 18 and 32 h. It was demonstrated that the higher time used for aging, the higher content of zeolite was obtained. It was determined that 86% LTA resulted in sample ZA5 within 30 h of aging, in comparison with 54% LTA in ZA4 with 24 h of aging. On the other hand, even though 32 h of aging was used for the synthesis of ZA6 with 66% LTA, the hydrothermal treatment time reduced to 16 h, promoting the decrease in LTA content in comparison that for ZA5. The longer aging time promoted the formation of a higher number of crystals; however, the crystalline products became smaller. Smaller particle sizes were obtained in samples ZA1 and ZA2 with hydrothermal treatment times of 24 h. Samples ZA3 and onwards demonstrated higher LTA particle sizes, which can be attributed to the reduction in hydrothermal treatment time. ZA5 was characterized by the highest content of LTA (86%) and crystallinity (94.53%). Thus, the interaction between aging and hydrothermal treatment times became critical for the synthesis of LTA zeolite in high content and crystallinity.

**Figure 4:**
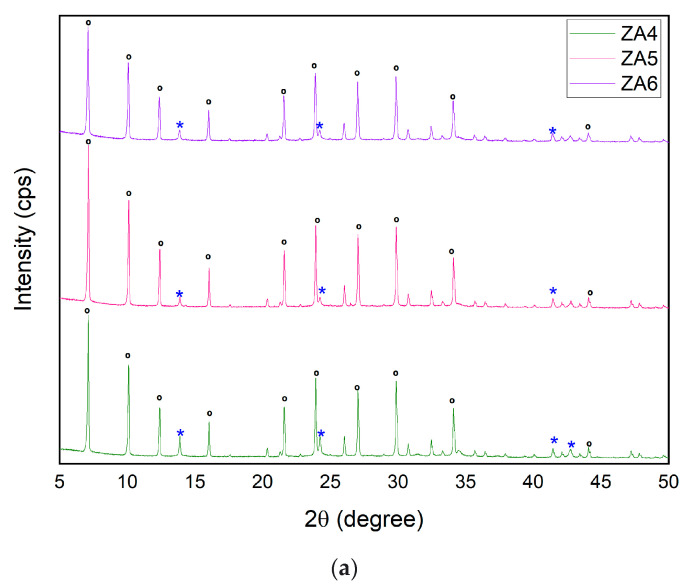

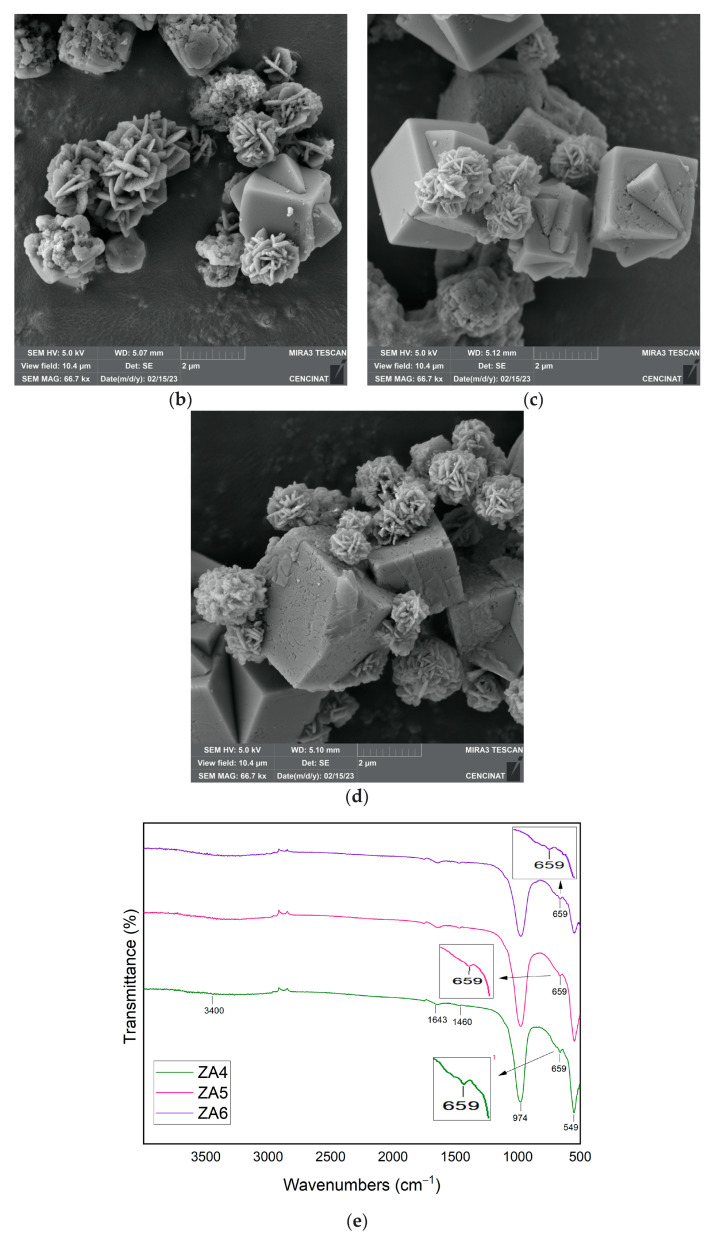
XRD patterns of synthesized zeolites ZA4, ZA5 and ZA6 (**a**), * sodalite and **°** LTA. SEM micrographs of (**b**) ZA4, (**c**) ZA5 and (**d**) ZA6. FTIR spectra for ZA4, ZA5 and ZA6 (**e**).

The XRD patterns of ZA4, ZA5 and ZA6 are depicted in Figure 4a. The reflections were found to be at the same 2θ position of the LTA zeolite pattern. ZA4 exhibited just four reflections belonging to the sodalite, at 2θ = 13.84° (011); 24.15° (112); 41.38° (088); and 42.55° (114); however, neither ZA5 nor ZA6 exhibited the reflection at 42.55° (114). The peaks of LTA were indexed to cubic crystal system and space group Fm-3c, with slight changes at unit cell parameters a[Å] = b[Å] = c[Å] = 24.62. The peaks of sodalite were indexed to cubic crystal system and space group Im-3m, with small changes at unit cell parameters a[Å] = b[Å] = c[Å] = 8.97. The intensity of the LTA and sodalite peaks of ZA5 were higher than those reflections observed in samples ZA4 and ZA6. The higher content of LTA zeolite was associated with fewer and lower-intensity sodalite peaks. Even though the same composition ratios were used in samples ZA4, ZA5 and ZA6, the content and crystallinity of LTA zeolite were also influenced by aging and hydrothermal treatment times. 

SEM micrographs of samples ZA4, ZA5 and ZA6 demonstrated the typical cubic morphology of LTA zeolite accompanied by lepispheres of sodalite (Figure 4b–d). The increase in aging time promoted a decrease in sodalite lepisphere size. The particle size of sodalite zeolite ranged between 1.3 and 1.4 µm in sample ZA5, and it remained around 1.1–1.4 µm for ZA6. Sample ZA5 was characterized by a well-defined cubic form, in comparison to both samples ZA4 and ZA6. The particle size of LTA zeolite in ZA5 ranged between 1.5 and 2.5 µm. However, when 32 h of aging time was used in ZA6, a not well-defined cubic form was obtained for LTA, and the particle size was between 2.5 and 3.3 µm. In this study, 30 h was optimal for the aging time, obtaining a high quantification and crystallinity of LTA zeolite.

The FTIR spectra for ZA4, ZA5 and ZA6 are depicted in Figure 4e. The absorption bands at 659 cm^−1^ denoted the existence of sodalite in ZA4, ZA5 and ZA6. The higher sodalite content (ZA4 > ZA6 > ZA5), the higher intensity of the absorption band at 659 cm^−1^. The absorption band at 1460 cm^−1^ found in samples ZA4 and ZA6 again suggested the existence of a hydroxy sodalite zeolite phase; however, it may have been below the limit of quantification because it was not identified by XRD analysis. 

#### 3.2.5. Effect of the Temperature of Calcination of Mining Tailing on the Synthesis of LTA Zeolite 

The synthesis of LTA zeolite was performed with and without calcination of the mining tailings. Initial assays demonstrated the convenience of using calcined mining tailings, which favored the production of LTA zeolite. Calcination of the raw material at high temperatures promoted the amorphization of mineralogical phases, and then the transformation into LTA zeolite was feasible. In this study, all of the synthesis assays were performed with alkaline fusion, which eased the dissolution of the silica and alumina, as well as the dissolution of other mineralogical phases [46]. A reduction in heavy metals content occurred after calcination of the mining tailings, which was attributed to the reduction in heavy metals chelated to organic matter. 

**Figure 5:**
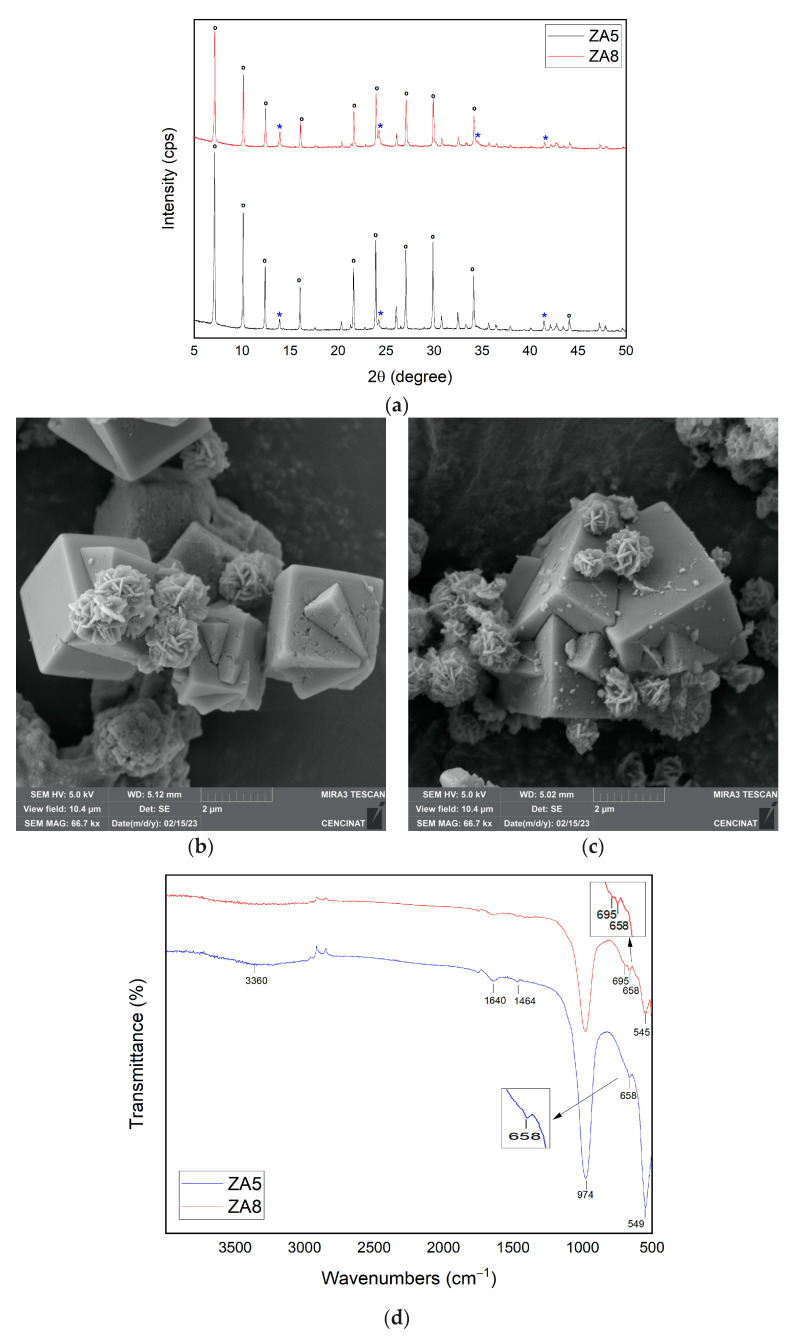
XRD patterns of synthesized zeolites ZA5 and ZA8 (**a**), * sodalite and **°** LTA. SEM micrographs of (**b**) ZA5 and (**c**) ZA8. FTIR spectra for ZA5 and ZA8 (**d**).

The XRD patterns of ZA5 and ZA8 are depicted in Figure 5a. The LTA peaks were indexed to the cubic crystal system and space group Fm-3c, with small changes in refined unit cell parameters a[Å] = b[Å] = c[Å] = 24.62. The peaks of sodalite were indexed to the cubic crystal system and space group Im-3m, with small changes in the refined unit cell parameters a[Å] = b[Å] = c[Å] = 8.97. Sodalite and LTA reflections were detected at the same 2θ positions discussed above, except for sodalite reflection at 37.43° (123), which was not found in ZA5. The intensity of the LTA and sodalite peaks decreased in sample ZA8 in comparison that in ZA5. A higher LTA zeolite content (86%) and crystallinity (94.53%) were associated with sample ZA5 in comparison to ZA8. The higher temperature that was used, the higher the content of LTA zeolite phase was obtained. Both samples ZA5 and ZA8 were synthesized under the same conditions, except that calcination of the mining tailings was performed at 800 °C and 600 °C, respectively. Thus, calcination of raw materials at high temperatures was an important stage of thermal pre-treatment that increased the solubility of the Al and Si species; thus, the higher the temperature, the higher the solubility of the phases and the easier formation of the LTA zeolite [47]. 

SEM micrographs revealed the typical well-defined cubic morphology for LTA zeolite and lepispheres for sodalite in both samples ZA5 and ZA8 (Figure 5b,c). The particle size of LTA zeolite in sample ZA5 ranged between 1.5 and 2.5 µm. On the other hand, when a lower calcination temperature of raw material was used, the particle size of LTA zeolite increased, as occurred in sample ZA8, with a particle size between 1.7 at 2.9 µm. Both samples ZA5 and ZA8 exhibited small sodalite particle sizes around 1.3–1.4 µm and 0.8–0.9 µm, respectively. Sample ZA5 had a higher content of LTA (86%) and crystallinity (94.53%) than sample ZA8, which contained 61% LTA and 81.47% crystallinity. 

The FTIR spectra for ZA5 and ZA8 are represented in Figure 5d. Sample ZA5 revealed a unique absorption band at 659 cm^−1^, suggesting a lower content of sodalite in comparison to sample ZA8 that exhibited two bands at 659 and 695 cm^−1^. The absorption band at 1464 cm^−1^ found in both samples ZA5 and ZA8 again suggested the existence of a hydroxy sodalite zeolite phase. The higher LTA content (86%) in sample ZA5 was obtained by the higher calcination temperature used for the mining tailing pre-treatment. The better alkali activation was obtained by calcining the precursor material; however, higher temperatures favored the formation of a hydroxy sodalite zeolite phase [48]. 

#### 3.2.6. Influence of Variations in SiO_2_/Al_2_O_3_ Ratio for the Synthesis of Zeolite LTA

The SiO_2_/Al_2_O_3_ ratio was relevant for the synthesis of LTA zeolite. Samples ZA6 and ZA7 were synthesized under the same operational conditions; however, the SiO_2_/Al_2_O_3_ ratio increased up to 13 for ZA7. The content of LTA zeolite in sample ZA7 was 99%, and the crystallinity was 93.18%. On the other hand, the lower SiO_2_/Al_2_O_3_ ratio yielded a lower LTA content (66%) and crystallinity (90.35%). Sample ZA5 was characterized by a lower SiO_2_/Al_2_O_3_ ratio, shorter aging time and longer hydrothermal treatment time. In ZA5, the content of LTA reached 86% and the crystallinity was around 94.53%. Alkalinity was also one of the most important parameters for the synthesis of LTA zeolite. The increase in alkalinity increased the rate of crystallization, both by nucleation and by crystalline growth. The higher concentration of reactive aluminosilicates allowed the phase change to LTA [41]. Higher amounts of aluminate, silicate and sodium (NaOH, NaAlO_2_) were used for synthesizing ZA7 in comparison to ZA6. Likewise, the increase in alkalinity also promoted a slight decrease in the particle size of the synthesized zeolite LTA, as occurred in sample ZA7.

**Figure 6:**
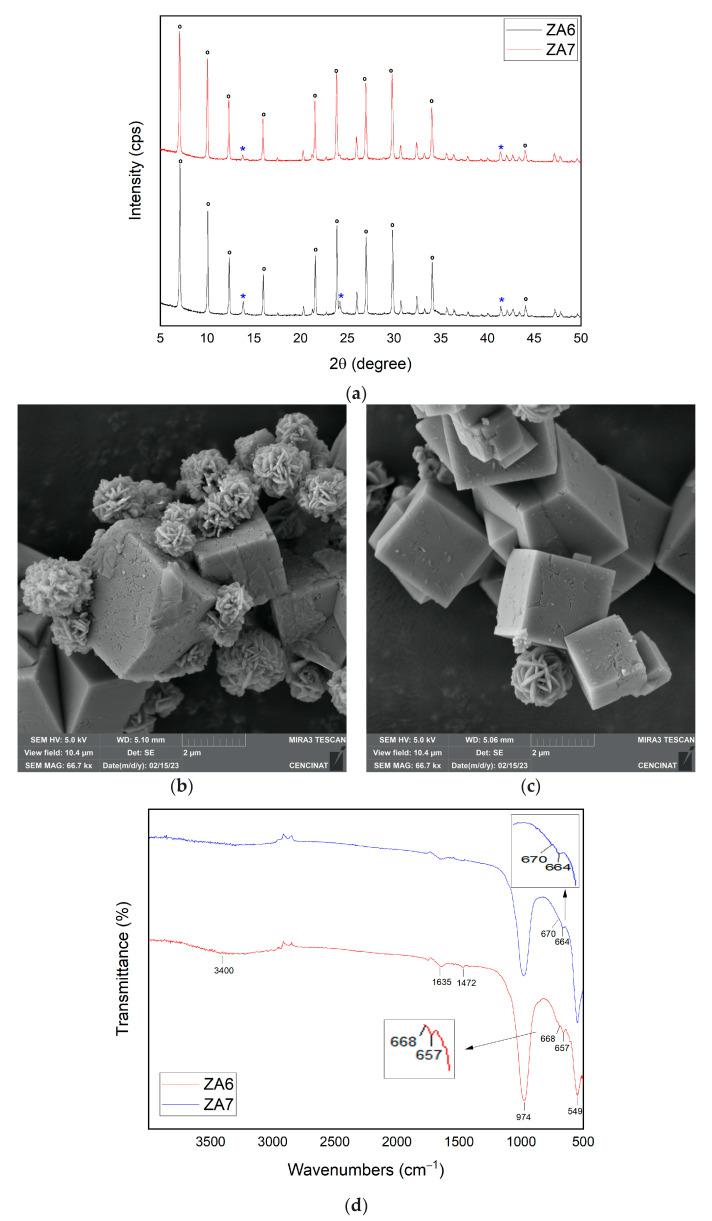
XRD patterns of synthesized zeolites ZA6 and ZA7 ((**a**), * sodalite and **°** LTA). SEM micrographs of (**b**) ZA6 and (**c**) ZA7. FTIR spectra for ZA6 and ZA7 (**d**).

The XRD patterns of both samples ZA6 and ZA7 are represented in Figure 6a. Sodalite reflections were detected at 2θ = 13.84° (011); 24.15° (112); and 41.38° (088) for ZA6; however, in ZA7 the reflection at 24.15° (112) was not found. The sodalite peaks were indexed to the cubic crystal system and space group Im-3m, with refined unit cell parameters a[Å] = b[Å] = c[Å] = 8.97. Both samples ZA6 and ZA7 revealed characteristic LTA reflections indexed to the cubic crystal system and space group Fm-3c, with modified refined unit cell parameters a[Å] = b[Å] = c[Å] = 24.64. The higher SiO_2_/Al_2_O_3_ ratio used in sample ZA7 (13), the higher the LTA content (99%) and crystallinity (93.18%). The lower the SiO_2_/Al_2_O_3_ ratio used in sample ZA6 (12), the lower LTA content (66%) and crystallinity (90.35%).

SEM micrographs revealed the cubic morphology of LTA and the lepispheres of sodalite in samples ZA6 and ZA7 (Figure 6b,c). A well-defined cubic form of LTA zeolite characterized sample ZA7 in comparison to ZA6. The particles sizes of LTA in sample ZA6 were around 2.5–3.3 µm, while the size of cubes of ZA7 were around 1.7–2.5 µm. Some cracks were also found in the faces of the cubic samples of ZA6 and ZA7. The particle size of sodalite ranged between 1.1 and 1.4 µm in sample ZA6, while size of cubes of sample ZA7 ranged between 1.7 and 2.1 µm. Previous reports associated beveled edges and cracks of LTA cubic crystals with a relatively higher alkalinity [49], as were found in ZA6 and ZA7. The SiO_2_/Al_2_O_3_ ratio differences between ZA6 and ZA7 can also explain the significant differences in morphology and particle size [40].

The FTIR spectra for both samples ZA6 and ZA7 are represented in Figure 6d. In ZA6, the absorption bands at 657 and 668 cm^−1^ evidenced the existence of sodalite. The absorption band at 1472 cm^−1^ found in ZA6 suggested the existence of a hydroxy sodalite zeolite phase. However, in ZA7, no absorption band around 1472 cm^−1^ was found, again suggesting a higher LTA zeolite content and crystallinity. 

### 3.3. Evaluation of Methylene Blue Adsorption Capacity of LTA Synthesized Zeolites

**Figure 7:**
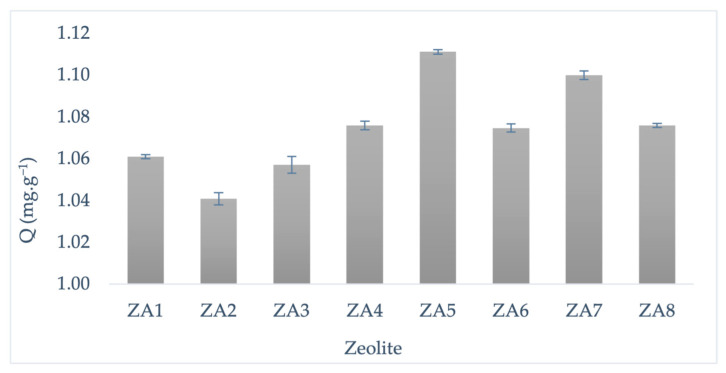
MB^+^ adsorption capacity onto synthesized LTA zeolites.

The methylene blue (MB^+^) adsorption capacity onto the synthesized LTA zeolites (ZA1–ZA8) is represented in Figure 7. The highest MB^+^ adsorption capacity was obtained onto ZA5 and ZA7, which are the two synthesized samples with the higher quantification and crystallinity of LTA zeolite obtained in this study. The higher content of LTA zeolite in both samples improved their adsorption performance, as the limited adsorptive properties of sodalite are well known [48]. The adsorption capacity of LTA zeolite followed the trend in crystallinity rather than LTA content. The LTA zeolite content was 86% in sample ZA5 and 99% in ZA7, while crystallinities of 94.53% and 93.18% were determined for ZA5 and ZA7, respectively. 

### 3.4. Support of Lithium Hydroxide Nanoparticles over LTA Zeolite

The chemical composition of lithium hydroxide nanoparticles over LTA zeolite (ZA-Li^+^) was determined by X-ray fluorescence (XRF) analysis (Table 4). SiO_2_ and Al_2_O_3_ were the main elements of the lithium hydroxide nanoparticles deposited over the LTA zeolite. There was 4.41 mg·g^−1^ of lithium doped on the ZA-Li^+^ zeolite. The low amount of Li^+^ doped could be attributed to the method of preparation used in this study. The production of lithium zeolite was carried out in accordance with a previous report where Li^+^ ions were easily exchangeable with Na^+^ ions [50].

**Figure 8:**
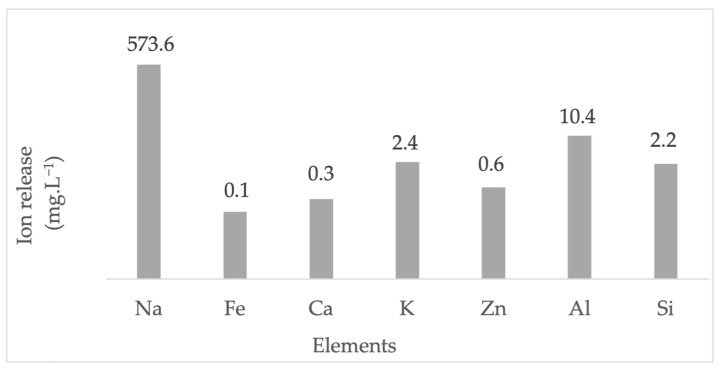
Elements released by the incorporation of lithium hydroxide nanoparticles over LTA zeolite. (Na, Fe, Ca, K and Zn were determined via AAS; Al and Si were determined using ICP-OES).

The ion exchange reaction between Li^+^ and ions from the LTA zeolite framework was verified by measuring the released elements in the exhausted solution. The ions released from zeolite during the incorporation of lithium are represented in Figure 8. The sodium content was determined to be high in solution, being the main exchange species. Low contents of aluminum, potassium, silicon, zinc, calcium and iron were found in the exhausted solution. The ionic radius has been reported to be the main constraint of the ion exchange reaction in the zeolite framework [50]. Monovalent lithium with a smaller ion radius (90 pm) easily occupied sites on LTA zeolite released by sodium (116 pm) > potassium (152 pm) > zinc (74 pm) > calcium (114 pm) > iron (65 pm). 

**Figure 9:**
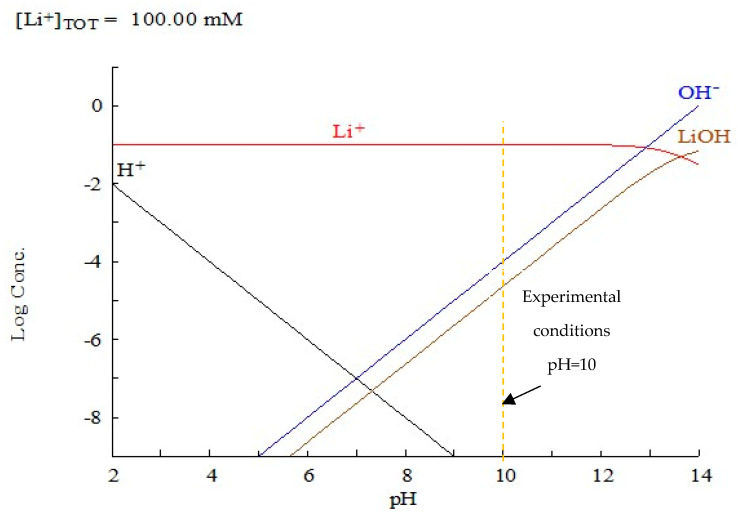
Logarithmic concentration Li^+^ species distribution diagram as a function of pH for the system LiCl (0.1 M Li^+^). Diagram was obtained using the HYDRA-MEDUSA numerical code.

The XRD patterns of ZA-Li^+^ and its parent ZA5 are depicted in Figure 9a. There XRD patterns of ZA-Li^+^ were similar to their parent ZA5; thus, the structure of LTA zeolite was not drastically affected by the incorporation of Li^+^ on LTA zeolite. LTA was indexed to the cubic crystal system and space group Fm-3c; after the incorporation of Li^+^ on LTA zeolite, the unit cell parameters were slightly reduced to a[Å] = b[Å] = c[Å] = 24.54. The peaks of sodalite were indexed to the cubic crystal system and space group Im-3m, with a modification of unit cell parameters a[Å] = b[Å] = c[Å] = 8.96. The structural contraction of Li^+^-exchanged LTA zeolite has also been previously reported [22]. The reflection of sodalite at 2θ: 41.38° (088) was not found in ZA-Li^+^; as well, the intensity of the reflections of LTA at 2θ: 29.82° (028) and 34.06° (466) decreased. Changes in peak intensities were observed in ZA-Li^+^, as previously reported in a lithium-exchanged zeolite. These results were attributed to the successful cationic exchange that promoted different occupations of cations in the pores, and the scattering power which is specific to each cation [22]. On the other hand, it was reported for zeolite 13X impregnated with LiCl and CaCl_2_ the appearance of new peaks between 30° and 35°, and peak intensities increased while the LiCl:CaCl_2_ salt ratios increased [51]. In our study, ZA-Li^+^ did not exhibit any new reflections, but a decrease in peak intensities was observed in the range between 30° and 35° in comparison to the parent ZA5. Chloride was not detected in the samples via FE-SEM, and the low content of lithium-incorporated ZA-Li^+^ did not allow for the identification of new crystalline phases using XRD; perhaps they were below the limit of quantification. However, small changes in the crystallographic parameters between ZA5 and ZA-Li^+^ suggested the partial incorporation of Li^+^ into the zeolite’s structure. For the parent zeolite ZA5, the *d*-spacing of (002) was 12.40 Å determined at 2q: 7.12. After, lithium was incorporated to ZA5 zeolite with a slight decrease in the *d*-spacing of (002) to 12.28 Å, occurring at 2q: 7.19. The slight changes determined for both the *d*-spacing and 2q position suggested a small structural rearrangement. The aluminum and silicon that were detected in the exhausted solution used for Li^+^ incorporation suggested the incorporation of Li^+^ by substitution of extra-framework Al^3+^ species, which promoted the substitution of the tetrahedral framework Si^4+^ species by Al^3+^. Electrostatic attraction is also another mechanism that could explain the incorporation of Li^+^ into the LTA zeolite framework, due to the natural negative charge of zeolites [12]. 

NaOH used to increase the pH to 10 during the incorporation of Li^+^ over the parent LTA zeolite (ZA5) promoted the formation of lithium hydroxide, which explained the presence of solid nanoparticles dispersed over the surface of ZA-Li^+^. Under these conditions, the coexistence of metal hydroxides Li(OH) at ≅10^−5^ (s) occurred in their ionic species Li^+^ at ≅10^−1^ (aq) and OH^−^ at ≅10^−4^ (aq) (Figure 9). Thus, under the experimental conditions, the existence of both forms, Li^+^ and Li(OH), allowed for the incorporation of Li^+^ by ionic exchange and electrostatic attraction as the main mechanisms, followed by precipitation of Li(OH) to a lesser extent. 

**Figure 10:**
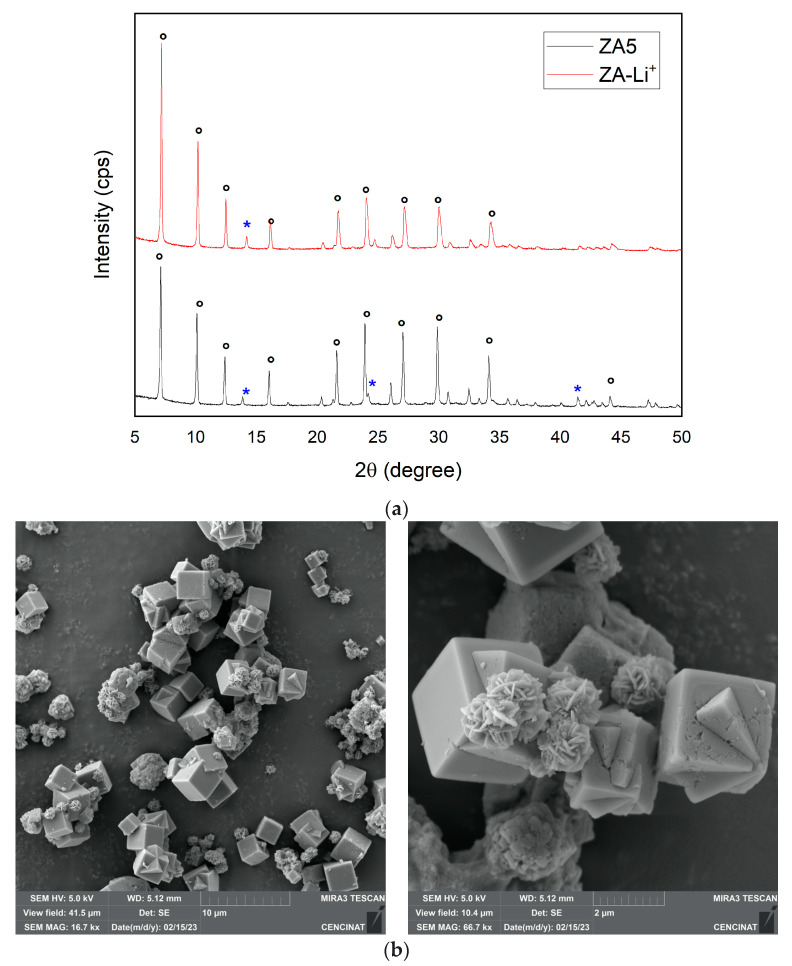

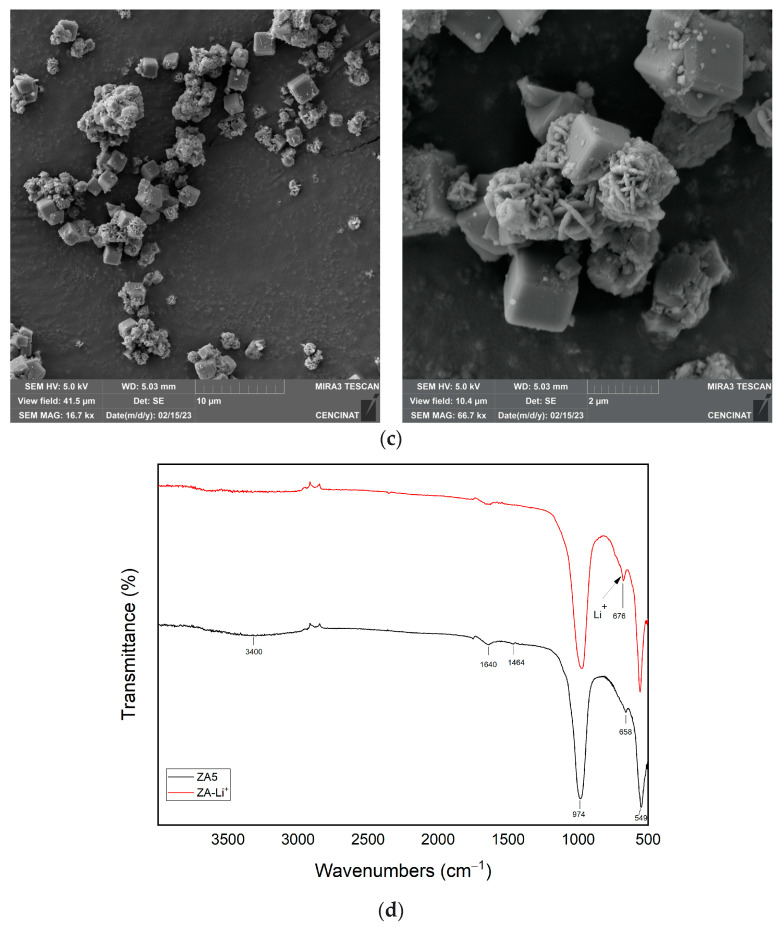
XRD patterns of the synthesized zeolite ZA5 and lithium hydroxide nanoparticles ZA-Li^+^ (**a**), * sodalite and **°** LTA. SEM micrographs of (**b**) ZA5 and (**c**) ZA-Li^+^. FTIR spectrum of ZA5 and ZA-Li^+^ (**d**).

SEM micrographs revealed small changes in the morphology of ZA5 when lithium was incorporated into ZA-Li^+^ (Figure 10b,c). The conventional smooth well-defined cubic morphology of LTA and the lepispheres of sodalite found in the parent ZA5 in ZA-Li^+^ turned out to be covered by crystal nanoparticles dispersed over the surface of the LTA cubic faces. The poor dispersion of lithium hydroxide nanoparticles over the cubic surface of LTA zeolite was evidenced in ZA-Li^+^, which is in accordance with the species distribution diagram mentioned above. The morphology of ZA-Li^+^ is different than a previously reported lithium-exchanged LTA zeolite that was characterized by a smooth surface zeolite [22], endorsing the incorporation of lithium hydroxide nanoparticles over LTA zeolite in this study.

The FTIR spectra for ZA5 and ZA-Li^+^ are depicted in Figure 10d. The characteristic absorption bands of sample ZA5 were registered at 658, 974 and 1640, and 3400 cm^−1^ was registered for sample ZA-Li^+^. Some variations in the position and intensity of those absorption bands at 676 cm^−1^ were observed for sample ZA-Li^+^. Cation movement, water–cation interactions and framework distortion were associated to a region with a range of 400–700 cm^−1^ [52]. Additionally, ZA-Li^+^ exhibited an intensity decrease in the broad absorption band at 3400 cm^−1^, suggesting a relationship between OH groups of zeolite and lithium cations, forming hydroxide groups [12]. The lithium hydroxide nanoparticles above were discussed based on the SEM photographs deposited over the LTA surface. Therefore, the mechanisms of incorporation of Li^+^ on the LTA framework can be explained in terms of ion exchange, isomorphic substitution, electrostatic attraction and lithium hydroxide precipitation. Thus, lithium hydroxide groups (≅LiOH) can become strategic sites for the adsorption of pollutants from water. 

**Figure 11:**
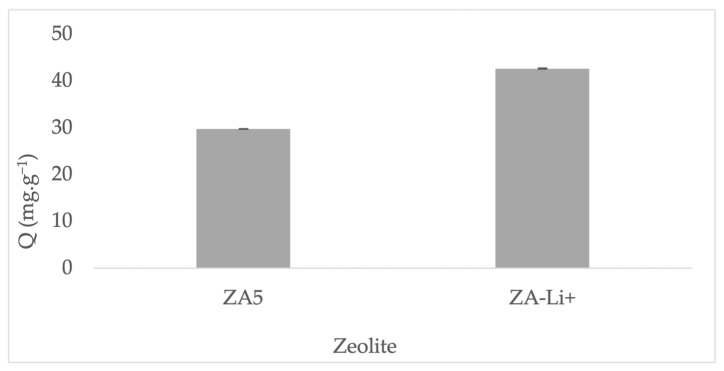
Methylene blue adsorption performance comparison between ZA5 and ZA-Li^+^.

The surface area of ZA5 (47.85 m^2^/g) was in accordance with other zeolites also obtained from mining tailings [17]. The surface area of sample ZA-Li^+^ (51.49 m^2^/g) increased 7% in comparison to its parent ZA5; similar results were obtained when a zeolite was modified by lithium through cationic exchange treatment. The pore volume also increased in ZA-Li^+^ (0.026 m^3^/g) in comparison to the parent sample ZA5 (0.024 m^3^/g). The slight increase in the pore volume can be explained in terms of ionic exchange of the zeolite framework, which promoted changes in the pore opening [22]. The increase in surface area was attributed to the creation of new adsorption sites, lithium hydroxide groups (≅LiOH), as this was verified by the adsorption capacity towards methylene blue as a sorbate model (Figure 11). The methylene blue adsorption capacity was 29.7 mg·g^−1^ for sample ZA5 and 42.6 mg·g^−1^ for sample ZA-Li^+^. An increase of 40% in the adsorption capacity towards methylene blue was achieved by ZA-Li^+^, exhibiting potential abilities for adsorption applications.

### 3.5. Adsorption Performance of the ZA-Li^+^

**Figure 12:**
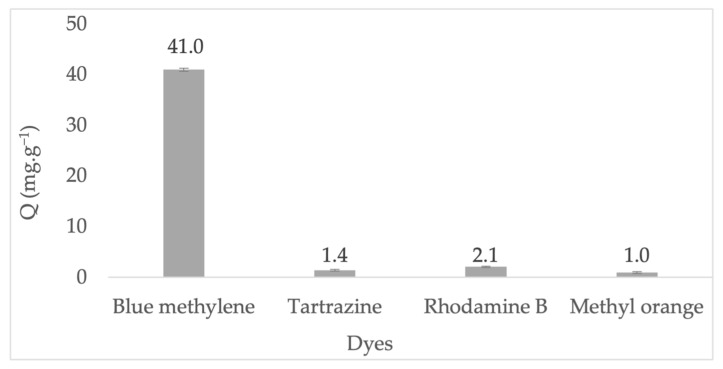
The adsorption performance of dyes onto ZA-Li^+^.

The adsorption performance of ZA-Li^+^ towards conventional dyes is depicted in Figure 12. The ZA-Li^+^ demonstrated the highest adsorption towards the cationic dyes methylene blue and rhodamine B. On the other hand, the adsorption towards the anionic dyes, tartazine and methyl orange, was almost negligible. The highest methylene blue adsorption onto ZA-Li^+^ was in accordance with that from a previous report when lithium allowed higher methylene blue adsorption onto a multiporous palygorskite [53]. Therefore, the highest affinity of ZA-Li^+^ by means of lithium hydroxide towards cationic dyes, particularly methylene blue, deserves a detailed study to explain the mechanisms that favor the strong attraction of MB^+^, particularly when the adsorption of MB^+^ was twenty times higher than rhodamine, which is also a cationic dye.

## 4. Conclusions

The synthesis of LTA zeolite with high crystallinity was performed using mining tailings as silica–alumina precursor. Conducting calcination at a high temperature as a mining tailings pre-treatment was favorable for the synthesis of LTA zeolite. Calcination also favored the elimination heavy metals chelated to organic matter, allowing for a suitable transformation into an LTA zeolite phase. The SiO_2_/Al_2_O_3_, Na_2_O/SiO_2_ and H_2_O/Na_2_O molar ratios were critical in obtaining a crystalline LTA zeolite product. Moreover, the operational conditions used throughout the synthesis were relevant: aging and hydrothermal treatment times. At optimized conditions, the synthesis of LTA zeolite with high crystallinity (94.53%) was performed. However, the sodalite zeolitic phase was a characteristic sub-product of the synthesis of LTA zeolite from low-cost precursors (mining tailing). The morphology of the zeolite synthesized was associated with the typical well-defined cubic form of LTA and lepispheres of sodalite. The adsorption capacity of methylene blue (MB^+^) increased when the crystallinity of the synthesized LTA zeolite increased. Lithium hydroxide nanoparticles were supported over the LTA zeolite (ZA-Li^+^), potentiating the adsorption properties of ZA-Li^+^. The ZA-Li^+^ sample demonstrated a higher affinity towards the adsorption of cationic dyes in comparison to anionic dyes. ZA-Li^+^ adsorbed methylene blue twenty-fold times more than for other cationic and anionic dyes. Thus, ZA-Li^+^ deserves further broad studies for the removal of methylene blue from wastewater, in order to know in detail the mechanisms involved. The advantage of this study is the use of mining tailings, which is a waste, to produce an LTA zeolitic material with high crystallinity. The added value of LTA zeolite is that tailings conventionally pollute the environment, while the synthesized LTA zeolite can remove pollutants from water, and have potential use for adsorption applications.

## Figures and Tables

**Table 1:** Chemical composition of mining tailings.

	Major Elements *									Minor Elements *				
ID	Al_2_O_3_ (%)	SiO_2_ (%)	P_2_O_5_ (%)	S (%)	K_2_O (%)	CaO (%)	Fe_2_O_3_ (%)	MnO (%)	BaO (%)	Cu ppm	Zn ppm	As ppm	Hg ppm	Pb ppm
^1^ BC	63.1	14.6	^3^ <lq	3.30	1.41	4.02	5.88	<lq	0.14	752	806	599	<lq	337
^2^ AC	57.2	12.2	0.27	2.44	1.11	3.30	5.70	0.15	<lq	0.11	0.19	0.15	0.03	0.10

* Elements were determined via X-ray fluorescence (XRF) analysis. ^1^ BC: before calcination, ^2^ AC: after calcination and ^3^ <lq: below the limit of quantification.

**Table 2:** Experimental conditions and products obtained from synthesis assays.

ID		Synthesis Conditions							Synthesized Phase ^b^	XRD Quantification (%)	LTA Crystallinity (%)
	Mine Tailings Status ^a^	Alkaline Fusion (°C)	Homogenization (h)	Aging (h)	Hydrothermal Treatment (h)	SiO_2_/Al_2_O_3_ Ratio	Na_2_O/SiO_2_ Ratio	H_2_O/Na_2_O Ratio			
SS1	NC	800	6	9	17	3	10	170	AMO	-	-
SS2	C	800	6	9	17	3	10	170	AMO	-	-
SS3	NC	800	6	17	24	7.4	2	170	AMO	-	-
SS4	C	800	6	17	24	8.5	2.4	40	SOD	93	-
									FIZ	7	
SS5	NC	800	6	17	24	7.4	3	40	CAN	89	-
									KOB	11	
SS6	C	800	1	24	24	8.5	3	40	SOD	83	-
									VLA	38	
SS7	NC	800	1	24	24	7.4	2.4	40	SOD	83	-
									RAM	17	
SS8	C	800	1	24	17	8.5	2	170	FAU	14	10.08
									SOD	23	
									RON	54	
									LTA	5	
ZA1	C	800	5	18	24	8	2	100	SOD	64	79.20
									LTA	36	
ZA2	C	800	5	18	24	10	2	100	SOD	63	81.64
									LTA	37	
ZA3	C	800	1	24	21	10	2	100	SOD	69	79.18
									LTA	31	
ZA4	C	800	1	24	21	12	2	100	SOD	46	82.87
									LTA	54	
ZA5	C	800	1	30	17	12	2	100	SOD	14	94.53
									LTA	86	
ZA6	C	800	1	32	16	12	2	100	SOD	34	90.35
									LTA	66	
ZA7	C	800	1	32	16	13	2	100	SOD	1	93.18
									LTA	99	
ZA8	C	600	1	30	17	12	2	100	SOD	39	81.47
									LTA	61	

^a^ C: calcined, NC: not calcined. ^b^ A: amorphous, SOD: sodalite, FIZ: fizelyte, CAN: cancrinite, KOB: kobellite, VLA: vladkrivovichevite, RAM: ramdohrite, FAU: faujasite-Na, LTA: Linde Type A, RON: rondorfite.

**Table 3:** Chemical composition of the synthesized LTA zeolites determined by X-ray fluorescence (XRF) analysis.

ID	Al_2_O_3_ (%)	SiO_2_ (%)	P_2_O_5_ (%)	S (%)	K_2_O (%)	CaO (%)	Fe_2_O_3_ (%)	ZnO (%)	SiO_2_/Al_2_O_3_	Si/Al
ZA1	19.6	25.5	<lq	0.4	0.1	1.5	1.9	0.1	1.3	1.1
ZA2	20.2	25.1	0.1	0.1	0.1	1.5	2.1	0.1	1.2	1.0
ZA3	19.1	27.2	<lq	0.4	0.1	1.6	1.9	0.2	1.4	1.2
ZA4	18.5	26.7	<lq	0.2	0.1	1.4	1.9	0.1	1.4	1.2
ZA5	18.1	26.2	0.1	0.1	0.1	1.6	2.1	0.2	1.5	1.2
ZA6	17.0	25.2	<lq	0.1	0.1	1.5	2.2	0.2	1.5	1.2
ZA7	17.1	23.8	<lq	0.1	0.1	1.4	2.1	0.2	1.4	1.2
ZA8	18.1	26.1	<lq	0.3	0.1	1.7	2.5	0.2	1.4	1.2

<1q: below the limit of quantification.

**Table 4:** Chemical composition of the synthesized ZA5 and modified zeolite ZA-Li^+^.

Major Elements *								
Al_2_O_3_ (%)	SiO_2_ (%)	P_2_O_5_ (%)	S (%)	K_2_O (%)	CaO (%)	Fe_2_O_3_ (%)	ZnO (%)	BaO (%)
18.1	26.1	ND	0.3	0.1	1.7	2.5	0.2	<lq
Trace elements *								
	Co_3_O_4_ ppm	CuO ppm	ZnO ppm	As_2_O_3_ ppm	Ba ppm	PbO ppm	MnO ppm	
	0.06	0.05	0.06	0.02	0.07	0.02	0.11	

* Major and trace elements were determined by X-ray fluorescence (XRF) analysis. <1q: below the limit of quantification.

## Data Availability

The data presented in this study are available on request from the corresponding author.
